# Association between presence of 20 or more natural teeth and all-cause, cancer-related, and cardiovascular disease-related mortality: Yamagata (Takahata) prospective observational study

**DOI:** 10.1186/s12903-020-01346-6

**Published:** 2020-12-02

**Authors:** Shigeo Ishikawa, Tsuneo Konta, Shinji Susa, Kenichi Ishizawa, Hitoshi Togashi, Yoshiyuki Ueno, Hidetoshi Yamashita, Takamasa Kayama, Mitsuyoshi Iino

**Affiliations:** 1 Department of Dentistry, Oral and Maxillofacial Plastic and Reconstructive Surgery, Faculty of Medicine, Yamagata University, 2-2-2 Iida-nishi, Yamagata, 990-9585 Japan; 2 Department of Public Health and Hygiene, Yamagata University Graduate School of Medicine, 2-2-2 Iida-nishi, Yamagata, 990-9585 Japan; 3 Department of Neurology, Hematology, Metabolism, Endocrinology and Diabetology, Yamagata University Faculty of Medicine, 2-2-2 Iida-nishi, Yamagata, 990-9585 Japan; 4 Yamagata University Health Administration Centre, 1-4-12 kojirakawa-machi, Yamagata, 990-8560 Japan; 5 Global Center of Excellence, Yamagata University School of Medicine, 2-2-2 Iida-nishi, Yamagata, 990-9585 Japan

**Keywords:** Tooth loss, Mortality, Prospective study, Observational study, Proportional-hazards model

## Abstract

**Background:**

Several studies have surveyed the relationship between the presence of ≥ 20 natural teeth and mortality. However, very few have evaluated this association over a long-term follow-up of more than ten years within a large population in Japan. This study aimed to prospectively confirm the associations between mortality and the presence of ≥ 20 natural teeth within a community-based population in Japan.

**Methods:**

A prospective observational study including 2208 participants aged ≥ 40 years was conducted in Takahata Town, Japan, between May 2005 and December 2016. All participants answered a self-administered questionnaire to provide their background characteristics, including their number of teeth. The participants were classified into two categories based on their self-reported number of teeth (< 20 and ≥ 20 teeth). Hazard ratios (HR) and 95% confidence intervals (CI) were calculated using Cox proportional-hazards regression model to assess risk factors for all-cause, cancer-, and cardiovascular disease-related mortality.

**Results:**

The total follow-up period was 131.4 ± 24.1 months (mean ± SD). After adjusting for covariates, the risk of all-cause mortality was significantly higher in the group with < 20 teeth than in those with ≥ 20 teeth (HR = 1.604, 95% CI 1.007–2.555, *p* = 0.047). However, the risk of cancer- and cardiovascular disease-related mortalities was not statistically significant between the two groups.

**Conclusion:**

In this study, participants with < 20 teeth had a significantly higher risk of all-cause mortality, although the difference was borderline significant. These results emphasize the importance of having ≥ 20 natural teeth for a healthy life expectancy.

## Background

In Japan, a political campaign for the preservation of ≥ 20 natural teeth at the age of 80 years has been conducted for more than 30 years by the Ministry of Health, Labour and Welfare [1, 2]. The outreach for the importance of keeping ≥ 20 natural teeth at the age of 80 years was successful, and the proportion of such individuals has been increasing in Japan [3]. Moreover, there has been growing evidence justifying the importance of having ≥ 20 natural teeth, as opposed to simply having several teeth regardless of the total number of teeth. Maintaining ≥ 20 teeth is crucial not just for mastication [4–6], but also beneficial for other aspects such as, decreasing the risk of onset of dementia [7], incident falls [8], and the requirement for nursing care [9].

**Fig. 1:**
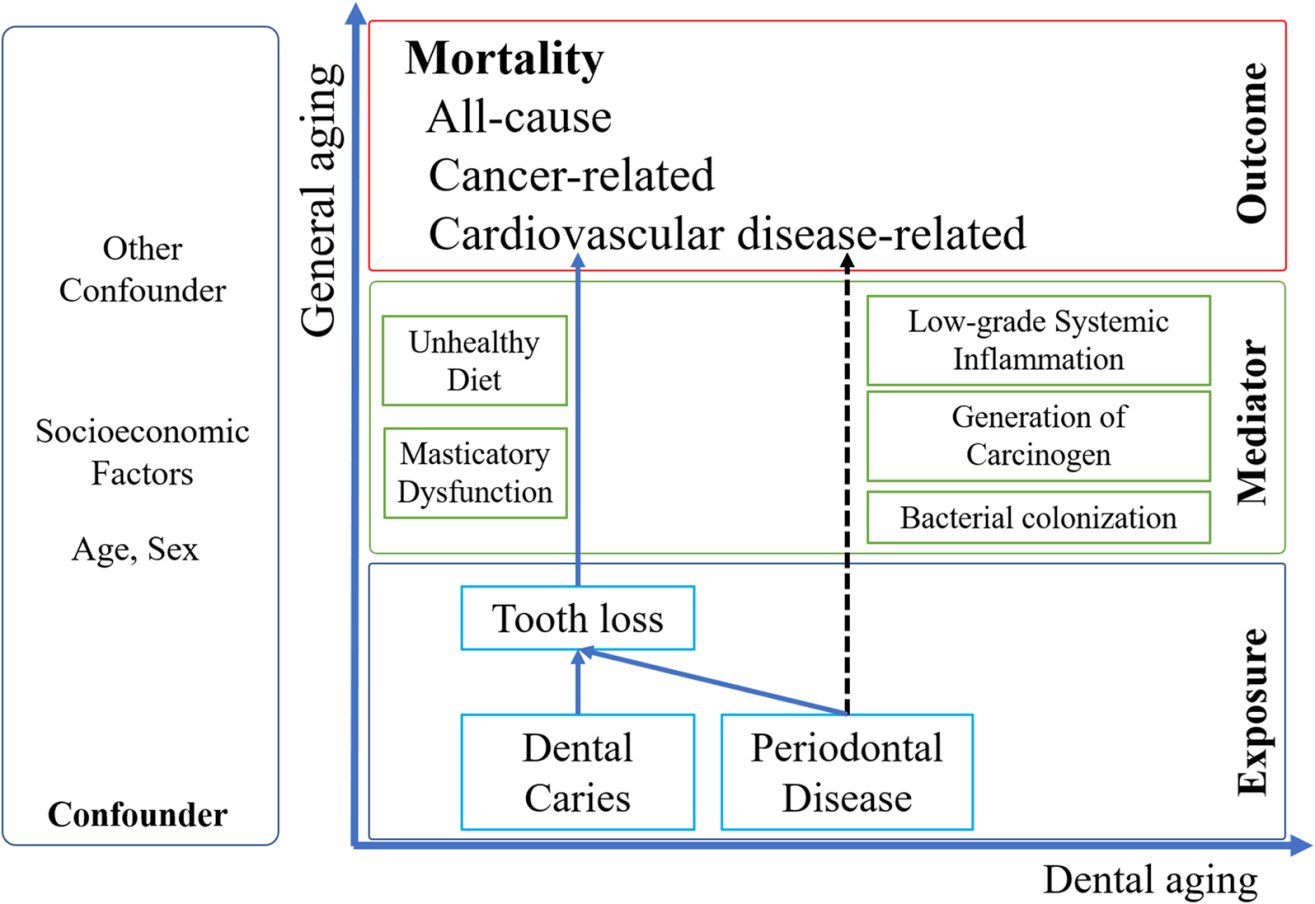
Causal graphs showing the relationship between tooth loss and all-cause, cardiovascular disease-related, or cancer-related mortality. This graph was modified from the original causal graphs created by Schwahn et al. [12]

The association between mortality and number of teeth has also attracted interest. Events such as tooth loss can lead to hypoactivity of the masticatory system, and, in turn, unhealthy diet patterns, which can negatively influence general health. Consequently, tooth loss has the potential to affect mortality [10–12]. The systemic inflammatory response in periodontal disease, a major cause for tooth loss, is a well-known risk factor for cardiovascular and malignant diseases [10–12]. Bacterial colonization and the generation of carcinogens such as nitrosamine due to periodontal disease are also frequently reported as potential risk factors for mortality, specifically cancer-related [10–12]. Based on the above hypotheses (Fig. 1), many prospective cohort studies worldwide have attempted to survey this relationship. Among these, several have surveyed the relationship between the presence of ≥ 20 natural teeth and mortality [13–16]. To the best of our knowledge, seven prospective studies on this topic have been published in Japan [17–23]. However, the participants of five of these studies were elderly people aged over 65 years [18–20, 22, 23], and only two studies were performed among community-dwelling people within the broad age range of Japanese adults [17, 21]. Furthermore, among these two studies, one had a relatively short follow-up period of less than 6 years [17].

Therefore, a prospective observational study was conducted with 2000 participants chosen from among community-dwelling people within the broad age range of Japanese adults, using data from a community-based cohort study, the Takahata study. The study was conducted over a period of more than ten years and was aimed at confirming the association between the presence of ≥ 20 natural teeth and mortality.

## Methods

### Study design and participants

This study was performed as part of the ongoing Molecular Epidemiological Study utilizing the Regional Characteristics of twenty first Century Centers of Excellence (COE) Program in Japan. This study was approved by the ethics committee of Yamagata University School of Medicine (No-2019-403).

The study participants were part of a community-based annual health check, in which residents of Takahata Town in Yamagata Prefecture, Japan, aged ≥ 40 years were invited to participate. The participants were in good general health; those who had subjective symptoms did not participate. Between May 2005 and December 2016, 2942 participants (1301 men, 1641 women) were enrolled. Fifty-nine participants were lost to follow-up after moving to other areas. Participants were followed-up for approximately 140 months and assessed for associations between their number of teeth and all-cause, cancer-related, and cardiovascular disease-related mortalities. Of the total number of participants, 734 were excluded due to incomplete data regarding their number of teeth. In total, 2208 participants were entered into the final statistical analysis.

### Measurements

At baseline, a postal survey in the form of a self-administered questionnaire was distributed among the participants to assess lifestyle factors, medical history, and oral health-related aspects, such as their number of teeth and dietary aspects, including alcohol consumption. This questionnaire has been used in previous studies [24, 25]. The number of teeth was assessed via a single-item question that was as follows: “How many teeth do you have now? (Fixed prostheses were counted; removable prostheses were not). The participants were divided into two groups based on self-reported number of teeth (< 20 teeth and ≥ 20 teeth). Note: People generally have 28 permanent teeth; some people have 29 to 32 permanent teeth (i.e., up to four wisdom teeth may be present).” Smoking status was classified into two categories: current smoker or non-smoker. Alcohol consumption was assessed using a brief self-administered diet history questionnaire [26], which inquired about the frequency of consumption of 58 food and beverage items. The total estimated intake of food and beverage items, energy, and selected nutrients was calculated using an ad hoc computer algorithm for the questionnaire, based on the Standard Tables of Food Composition in Japan. The validity of this questionnaire has already been assessed, and it has been widely used in epidemiological nutrition studies in Japan [26–28]. Perceived mental stress was assessed via a single question: “Have you experienced dissatisfaction, distress, a hard time, or stress associated with life during the last month?” For educational status assessment, the participants were divided into three groups based on their age during their highest educational qualification: the high educational status group (> 19 years of age), the middle educational status group (≤ 18 years of age, > 15 years of age), and the low educational status group (≤ 15 years of age). This categorization was based on the fact that people generally graduate from junior high school at 15 years of age and from high school at 18 years of age in Japan. The low educational status group and the middle educational status group consisted of people who had graduated from junior high school and senior high school respectively, while the high educational status group consisted of those who had achieved any college or higher education.

Laboratory parameters were obtained at the annual health check site during baseline. Hypertension was defined by a systolic/diastolic blood pressure ≥ 140/90 mmHg (Japanese Society of Hypertension) [29], and/or treatment with antihypertensive medications [30]. The presence of diabetes mellitus (DM) was defined as fasting plasma glucose level ≥ 126 mg/dL, hemoglobin A1c level ≥ 6.5% (Japanese Diabetes Society), or treatment with antidiabetic medications.

Details about the participants’ death and cause of death were collected from the Summary Report of Annual Vital Statistics of Japan from the Ministry of Health, Labour and Welfare for the town of Takahata, and from the cancer registration data of the Yamagata prefecture.

### Statistical analyses

The distribution of characteristics was analyzed using Mann–Whitney U-test and the chi-squared test for quantitative and qualitative variables, respectively. Hazard ratios (HR) and 95% confidence intervals (CI) were calculated using Cox proportional-hazards regression model to assess risk factors for all-cause, cancer-, and cardiovascular disease-related mortality. In the multivariate-adjusted model, HR was adjusted for age, sex, smoking habit, alcohol consumption, educational status, hypertension, DM, and perceived mental stress. These adjusted factors for the Cox regression model have also been used in previous studies that surveyed the association between number of teeth and mortality [13–15, 19, 21].

To examine the independent association between all-cause mortality in the groups with either < 20 or ≥ 20 teeth and several continuous or categorical parameters, HRs and 95% CI for the risk of all-cause mortality were calculated using Cox proportional-hazards regression analysis. A forced-entry method for the Cox proportional-hazards model with was used.

To examine whether the association between number of teeth and mortality differed according to background characteristics, subgroup analyses were performed using Cox proportional-hazards regression model, and the adjusted HR was calculated in each subgroup. Age, sex, body mass index (BMI), smoking habit, alcohol consumption, educational status, hypertension, DM, and perceived mental stress were also adjusted for in the subgroup analyses.

Survival curves were drawn to examine the relationships between number of teeth and all-cause, cancer-, and cardiovascular disease-related mortality. These were based on Cox regression models adjusted for age, sex, BMI, smoking habit, alcohol consumption, educational status, hypertension, DM, and perceived mental stress. Statistical significance was set at *p* < 0.05. Statistical analyses were performed with SPSS version 20.0 (IBM Corp., Armonk, NY, USA).

## Results

The total follow-up period for the participants was 131.4 ± 24.1 months (mean ± SD). For the participants who died, the follow-up period was 82.8 ± 24.1 months, and for the ones who survived until the end of our study, the period was 137.4 ± 12.3 months. During follow-up, a total of 219 deaths were recorded. Of these, the deaths that were cancer- and cardiovascular disease-related were 82 and 55, respectively. From a total of 2208 participants, 1989 participants (90.1%) were censored; 42 of 1989 participants were lost to follow-up after moving to another area. The distribution of the clinical parameters in each group (< 20 and ≥ 20 natural teeth) are shown in Table 1. Participants with < 20 teeth were significantly older than those with ≥ 20 teeth. Alcohol consumption was significantly less in the participants with < 20 teeth than in those with ≥ 20 teeth. BMI was not significantly different between the two groups. The chi-squared test also revealed significant differences in the distribution of parameters between the two groups, which included sex, hypertension, DM, and stressful life events during the last month. There were no significant differences in the distribution of educational status and smoking habits between the two groups.

**Table 1:** Participant characteristics

		Number of teeth				*p* value^†^
		<20		≥20		
		Average	SD	Average	SD	
	Total	*n* = 793		*n* = 1407		
Age (years)	2200	68.0	8.3	58.8	9.5	<0.001*
	Total	*n* = 779		*n* = 1392		
BMI (kg/m^2^)	2171	23.4	4.0	23.4	3.2	0.275
		*n* = 670		*n* = 1257		
Alcohol consumption (g/day)	1927	11.3	23.6	13.3	26.0	0.004

		Number of teeth				*p* value^‡^
		<20		≥20		
Variable		*n*	%	*n*	%	
Sex	Male	374	47.0	586	41.9	0.012*
	Female	421	53.0	827	58.5	
	Total	795		1413		
Educational status	High	121	18.7	220	18.7	1.000
	Middle	364	56.3	664	56.4	
	Low	161	24.9	293	24.9	
	Total	646		1177		
Hypertension	No	255	45.5	594	60.9	<0.001*
	Yes	306	54.5	381	39.1	
	Total	561		975		
Diabetes mellitus	No	518	92.3	934	95.8	0.005*
	Yes	43	7.7	41	4.2	
	Total	561		975		
Current smoker	No	555	81.9	1022	80.8	0.584
	Yes	123	18.1	243	19.2	
	Total	678		1265		
Perceived mental stress	Never	42	5.3	62	4.4	<0.001*
	Not very often	244	31.0	312	22.2	
	Sometimes	409	52.0	817	58.1	
	Most of the time	91	11.6	214	15.2	
	Total	786		1405		

SD, standard deviation; BMI, body mass index

^†^*p* value by Mann–Whitney U-test

^‡^*p* value by chi-squared test

Table 2 shows unadjusted and adjusted HRs. In the unadjusted model, the risk of all-cause, cancer-related, and cardiovascular disease-related mortality was significantly higher in the group with < 20 teeth than in the group with ≥ 20 teeth (all-cause mortality: HR = 2.658, 95% CI 2.030–3.479, *p* < 0.001; cancer-related mortality: HR = 2.127, 95% CI 1.378–3.283, *p* = 0.001; cardiovascular disease-related mortality: HR = 2.760, 95% CI 1.609–4.735, *p* < 0.001). In the adjusted model, the risk for all-cause mortality was significantly higher in the group with < 20 teeth than in the group with ≥ 20 teeth (HR = 1.604, 95% CI 1.007–2.555, *p* = 0.047). However, the CI of the HR was very close to the null value and the difference was borderline significant. The risk of cancer-related and cardiovascular disease-related mortality was not significantly higher in the group with < 20 teeth than in the group with ≥ 20 teeth in the adjusted model.

**Table 2:** Cox regression analysis: Associations between the number of teeth and all-cause mortality

Variable		Unadjusted			Adjusted		
		HR	95% CI	*p* value	HR	95% CI	*p* value
All-cause mortality							
Number of teeth ≥ 20	(*n* = 90/1413)	Reference			Reference		
< 20	(*n* = 129/795)	2.658	(2.030–3.479)	< 0.001*	1.604	(1.007–2.555)	0.047*
Cancer-related mortality							
Number of teeth ≥ 20	(*n* = 38/1413)	Reference			Reference		
< 20	(*n* = 44/795)	2.127	(1.378–3.283)	0.001*	1.719	(0.798–3.700)	0.166
Cardiovascular disease-related mortality							
Number of teeth ≥ 20	(*n* = 22/1413)	Reference			Reference		
< 20	(*n* = 33/795)	2.760	(1.609–4.735)	< 0.001*	1.289		0.570

Adjusted for age, sex, body mass index, smoking habit, alcohol consumption, educational status, hypertension, diabetes mellitus, and perceived mental stress

*HR* hazard ratio, *CI* confidence interval

^*^Statistically significant (*p* < 0.05)

Table 3 shows adjusted HRs for all variables associated with all-cause mortality. Having < 20 teeth, age, sex (male), smoking habit, and fewer episodes of perceived mental stress were independent significant risk factors for all-cause mortality.

**Table 3:** Adjusted hazard ratios and 95% confidence intervals for variables associated with all-cause mortality

Variable		Adjusted HR	(95% CI)	*p *value
Tooth number	(< 20 teeth vs ≥ 20 teeth)	1.604	1.007–2.555	0.047*
Age (years)	(per 1 year increase)	1.091	1.059–1.123	< 0.001*
Sex	(female vs male)	0.434	0.261–0.719	0.001*
BMI (kg/m^−2^)	(per 1 kg/m^2^ increase)	0.955	0.898–1.017	0.150
Smoking habit	(Yes vs no)	1.916	1.128–3.257	0.016*
Alcohol consumption (g/day)	(per 1 g/day increase)	0.998	0.989–1.007	0.640
Educational status	(High vs low)	0.938	0.557–1.527	0.798
	(Middle vs low)	0.701	0.365–1.345	0.285
Hypertension	(Yes vs no)	1.199	0.770–1.865	0.422
Diabetes mellitus	(Yes vs no)	1.779	0.960–3.296	0.067
Perceived mental stress	(Not very often vs never)	0.467	0.224–0.972	0.042*
	(Sometimes vs never)	0.495	0.247–0.992	0.047*
	(Most of the time vs never)	0.648	0.255–1.643	0.360

Adjusted for age, sex, body mass index, smoking habit, alcohol consumption, educational status, hypertension, diabetes mellitus, and perceived mental stress

*HR* hazard ratio, *CI* confidence interval

^*^statistically significant (*p* < 0.05)

Table 4 shows adjusted HRs for all variables associated with all-cause mortality in participants having < 20 teeth; age and sex (male) were significant risk factors for all-cause mortality.

**Table 4:** Adjusted hazard ratios and 95% confidence intervals for variables associated with all-cause mortality in the group having < 20 teeth

Variable		Adjusted HR	(95% CI)	*p* value
Age (years)	(per 1 year increase)	1.118	(1.017–1.167)	< 0.001*
Sex	(female vs male)	0.331	(0.107–0.645)	0.001*
BMI (kg/m^−2^)	(per 1 kg/m^2^ increase)	0.979	0.901–1.065	0.627
Smoking habit	(Yes vs no)	1.356	0.659–2.788	0.408
Alcohol consumption (g/day)	(per 1 g/day increase)	1.001	0.988–1.014	0.897
Educational status	(High vs low)	1.251	0.657–2.379	0.496
	(Middle vs low)	0.984	0.426–2.272	0.971
Hypertension	(Yes vs no)	1.482	0.821–2.673	0.192
Diabetes mellitus	(Yes vs no)	1.328	0.588–2.995	0.495
Perceived mental stress	(Not very often vs never)	0.453	0.179–1.146	0.095
	(Sometimes vs never)	0.522	0.215–1.268	0.151
	(Most of the time vs never)	1.043	0.325–3.351	0.943

Adjusted for age, sex, BMI, smoking habit, alcohol consumption, educational status, hypertension, diabetes mellitus, and perceived mental stress

*HR* hazard ratio, *CI* confidence interval, *BMI* body mass index

^*^Statistically significant (*p* < 0.05)

Table 5 shows adjusted HRs for all variables associated with all-cause mortality in participants having ≥ 20 teeth; age and smoking habit were significant risk factors for all-cause mortality.

**Table 5:** Adjusted hazard ratios and 95% confidence intervals for variables associated with all-cause mortality in the group having ≥ 20 teeth

Variable		Adjusted HR	(95% CI)	*p *value
Age (years)	(per 1 year increase)	1.065	1.023–1.109	0.002*
Sex	(female vs male)	0.630	0.271–1.465	0.283
BMI (kg/m^−2^)	(per 1 kg/m^2^ increase)	0.912	0.815–1.022	0.112
Smoking habit	(Yes vs no)	3.711	1.642–8.387	0.002*
Alcohol consumption (g/day)	(per 1 g/day increase)	0.995	0.980–1.010	0.532
Educational status	(High vs low)	0.752	0.342–1.654	0.479
	(Middle vs low)	0.500	0.170–1.471	0.208
Hypertension	(Yes vs no)	0.756	0.351–1.626	0.474
Diabetes mellitus	(Yes vs no)	2.555	0.933–6.994	0.068
Perceived mental stress	(Not very often vs never)	0.465	0.128–1.690	0.245
	(Some of the time vs never)	0.398	0.117–1.355	0.140
	(Most of the time vs never)	0.355	0.070–1.812	0.213

Adjusted for age, sex, BMI, smoking habit, alcohol consumption, educational status, hypertension, diabetes mellitus, and perceived mental stress

*HR* hazard ratio, *CI* confidence interval, *BMI* body mass index

^*^Statistically significant (*p* < 0.05)

The results of the subgroup analyses for associations between the number of teeth and all-cause mortality are shown in Table 6. The HRs for all-cause mortality were significantly higher in the group with < 20 teeth than in the group with ≥ 20 teeth among subgroups such as male, age > 65 years, no smoking habits, hypertension, and non-diabetics. In particular, non-smokers with < 20 teeth had a 2.414-fold risk for all-cause mortality compared to non-smokers with ≥ 20 teeth. Further, non-diabetics with < 20 teeth had a 1.715-fold risk for all-cause mortality compared to non-diabetics with ≥ 20 teeth. The interaction between number of teeth and age (*p* = 0.053), and between number of teeth and smoking (*p* = 0.055), showed borderline significance. However, the interactions between number of teeth and DM, and between number of teeth and hypertension, were not significant (DM: *p* = 0.198, hypertension: *p* = 0.1421).

**Table 6:** Subgroup analysis: Associations between the number of teeth and all-cause mortality

		Number of teeth			
		≥ 20	< 20		
Variable (number of cases/number of samples)		HR	HR	(95% CI)	*p* value
Sex					
Men	153/960	1.0	1.775	(1.012–3.114)	0.045*
Women	66/1248	1.0	1.382	(0.565–3.380)	0.479
Age					
< 65 years	48/1241	1.0	1.031	(0.411–2.583)	0.948
≥ 65 years	170/959	1.0	3.126	(1.651–5.922)	< 0.001*
BMI					
≤ 18.5	16/82	1.0	0.908	(0.155–5.314)	0.915
18.5< , ≤ 25	136/1477	1.0	1.527	(0.859–2.711)	0.149
25<	61/612	1.0	2.149	(0.800–5.771)	0.129
Smoking habit					
No	150/1577	1.0	2.412	(1.364–4.266)	0.002*
Yes	48/366	1.0	0.673	(0.263–1.723)	0.409
Alcohol consumption					
< 20 g	143/1524	1.0	1.687	(0.976–2.916)	0.061
≥ 20 g	40/403	1.0	1.894	(0.719–4.992)	0.196
Educational status					
High	33/341	1.0	3.229	(0.922–11.302)	0.067
Middle	106/1028	1.0	1.809	(0.967–3.385)	0.064
Low	45/454	1.0	1.218	(0.487–3.043)	0.673
Hypertension					
Yes	82/687	1.0	2.686	(1.285–5.614)	0.009*
No	66/849	1.0	1.049	(0.543–2.025)	0.887
Diabetes mellitus					
Yes	16/84	1.0	0.999	(0.244–4.088)	0.999
No	132/1452	1.0	1.715	(1.035–2.841)	0.036*
Perceived mental stress					
Never	18/104	1.0	2.816	(0.373–21.271)	0.316
Not very often	61/556	1.0	1.214	(0.502–2.937)	0.667
Some of the time	113/1226	1.0	1.628	(0.826–3.21)	0.159
Most of the time	24/305	1.0	3.109	(0.658–14.692)	0.152

Adjusted for age, sex, BMI, smoking habit, alcohol consumption, educational status, hypertension, diabetes mellitus, and perceived mental stress

*HR* hazard ratio, *CI* confidence interval, *BMI* body mass index

^*^statistically significant (*p* < 0.05)

**Fig. 2:**
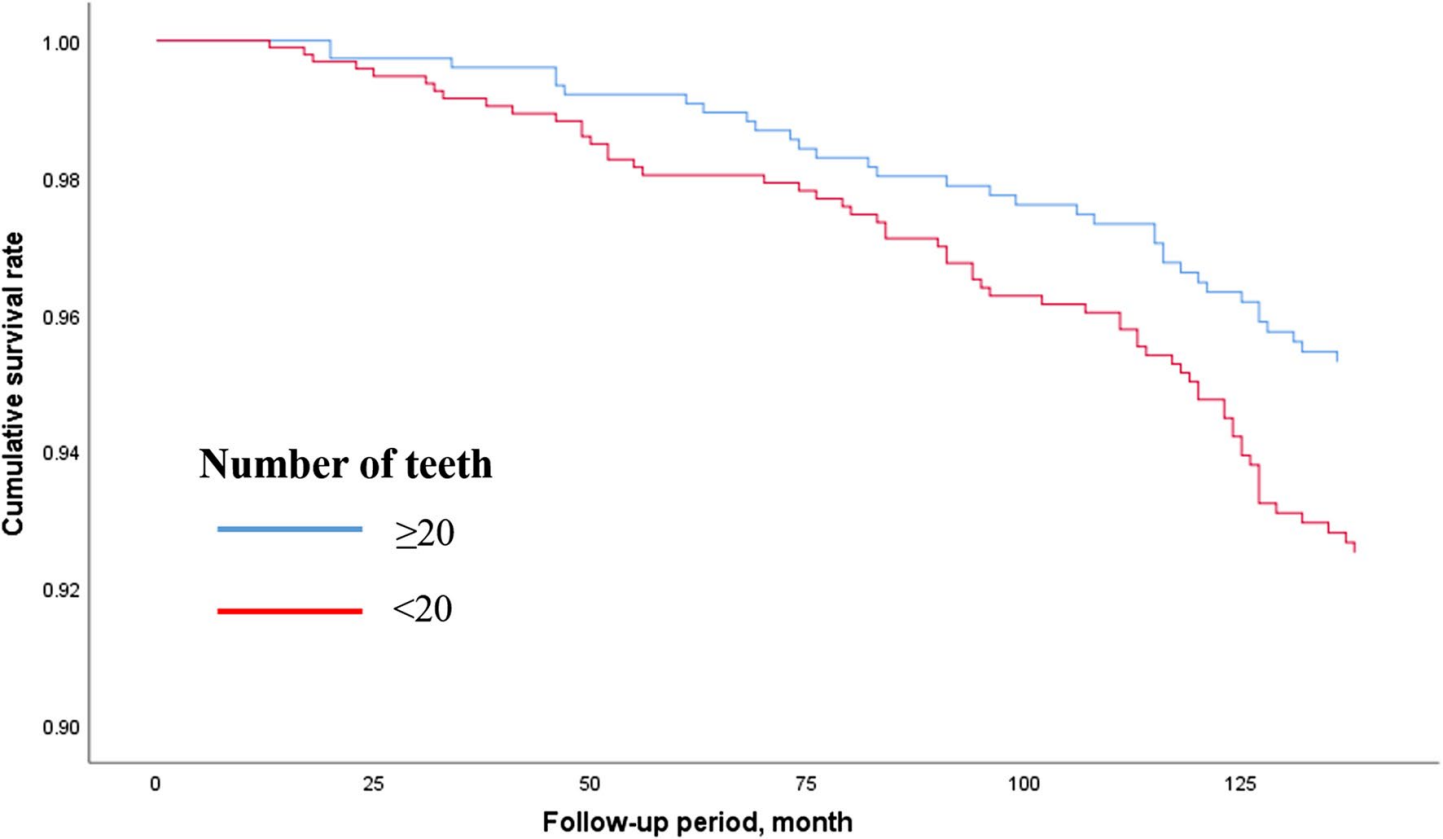
Survival curve for all-cause mortality according to number of teeth based on Cox regression models adjusted by age, sex, BMI, smoking habit, alcohol consumption, educational status, hypertension, diabetes mellitus, and perceived mental stress. *BMI* Body mass index

**Fig. 3:**
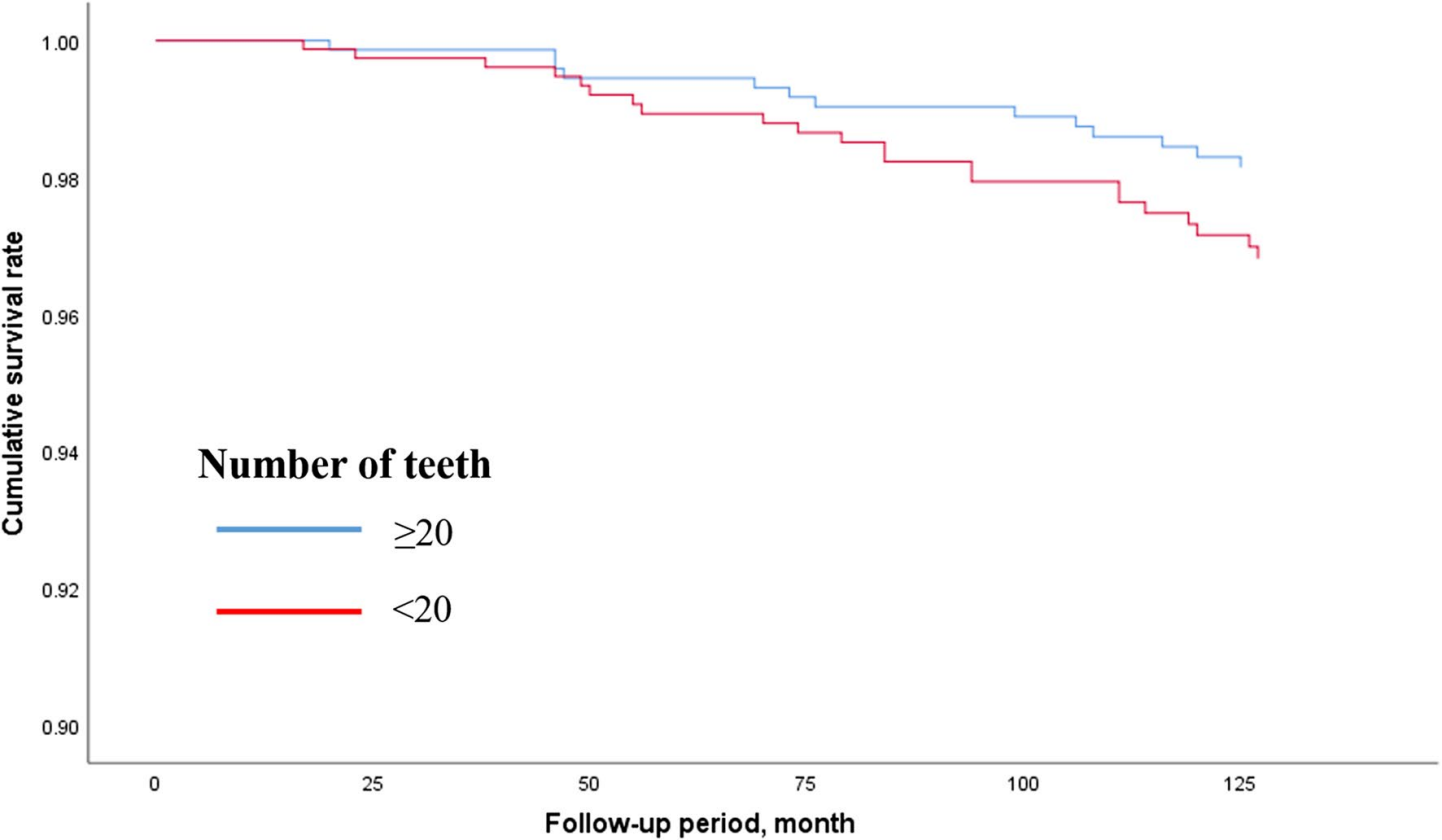
Survival curve for cancer-related mortality according to number of teeth based on Cox regression models adjusted by age, sex, BMI, smoking habit, alcohol consumption, educational status, hypertension, diabetes mellitus, and perceived mental stress. *BMI* Body mass index

**Fig. 4:**
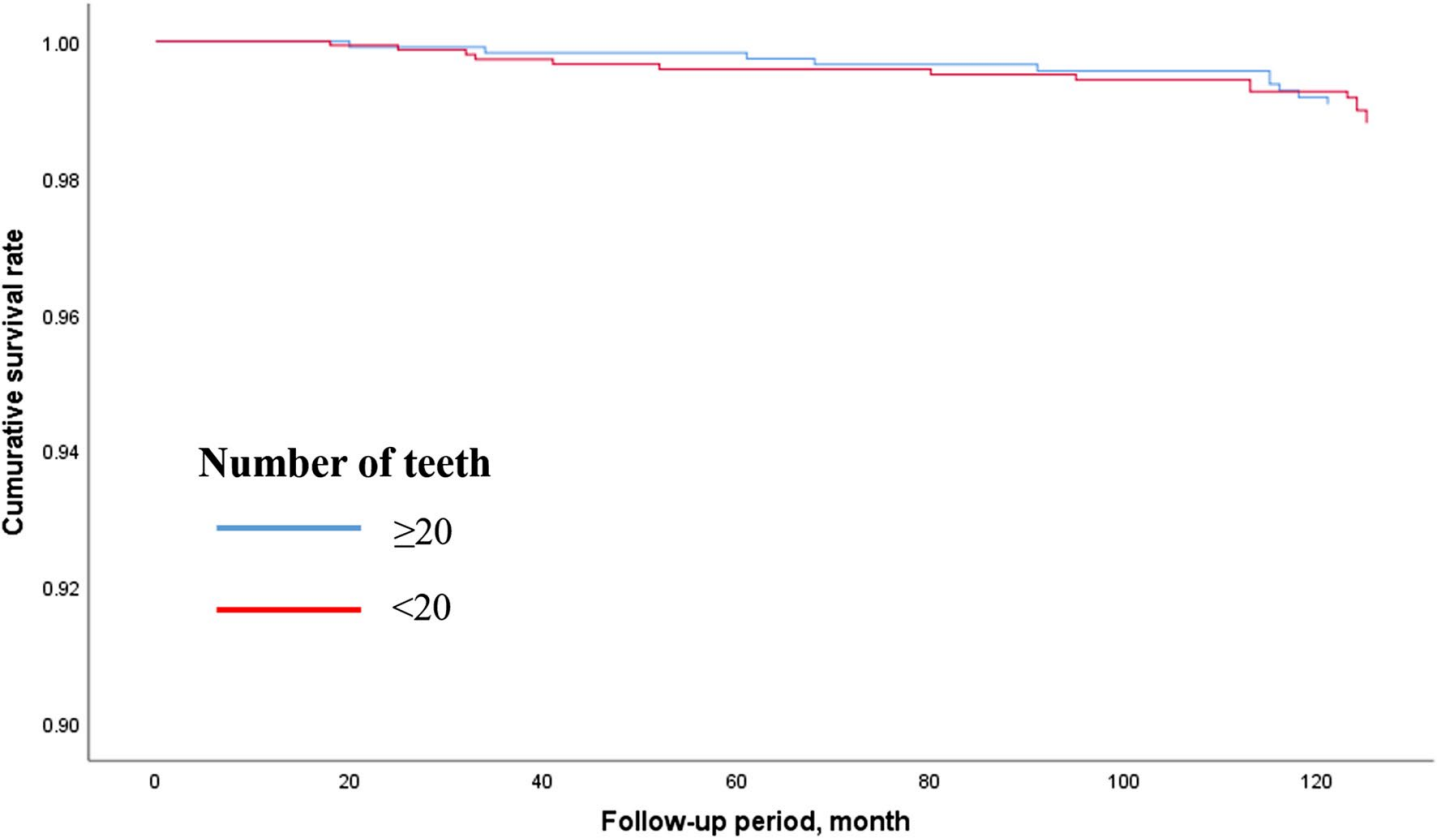
Survival curve for cardiovascular disease-related mortality according to number of teeth based on Cox regression models adjusted by age, sex, BMI, smoking habit, alcohol consumption, educational status, hypertension, diabetes mellitus, and perceived mental stress. *BMI* Body mass index

Figures 2, 3 and 4 show the adjusted survival curves based on Cox regression model for the all-cause, cancer-, and cardiovascular disease-related mortality between participants having < 20 and ≥ 20 teeth. The survival rate for all-cause mortality was evidently higher in the participants having ≥ 20 teeth than in the participants having < 20 teeth.

## Discussion

In this study, we prospectively investigated the associations between the number of teeth and mortality in a region in Japan. We observed that people with < 20 natural teeth have a significant risk for all-cause mortality. To the best of our knowledge, this study is one of the largest studies covering a broad age range in the Japanese population, with one of the longest follow-up periods. This makes our study note-worthy.

The mechanism underlying the association between the presence of < 20 natural teeth and the risk for all-cause mortality is unclear; however, some hypotheses have been suggested. Events such as tooth loss, lead to hypoactivity of the masticatory system and ultimately, insufficient nutrient intake, which can have a negative influence on general health [13, 31]. Yoshihara et al. reported that participants with < 20 teeth had a significantly lower total protein, animal protein, sodium, vitamin D, vitamin B1, vitamin B6, niacin, and pantothenic acid intake than participants with ≥ 20 teeth [32]. Furthermore, subjective mastication difficulties, particularly when eating hard food, begin to appear when there are < 20 teeth in the mouth [33, 34]. Tooth loss leads to reduced nutrient intake, which, in turn, may be associated with unhealthy conditions, such as being underweight or developing certain diseases [25, 32, 35, 36]. Ultimately, these factors may be linked to increased mortality.

Moreover, several studies have revealed the associations between poor masticatory function as a result of < 20 teeth and poor cognitive function [7], risk of incident falls [8], and requirement for nursing care [9], of which are associated with mortality. Furthermore, the association between tooth loss, poor masticatory function, and mortality has also been proven epidemiologically [37]. Therefore, the association between < 20 teeth and risk of all-cause mortality is justified.

We would also like to discuss the underlying mechanisms that associate tooth loss with the risk of all-cause mortality, based on the causes for tooth loss. Although tooth loss is primarily caused by dental caries or periodontal disease, tooth loss due to periodontitis must affect mortality differently than that due to dental caries. Periodontal disease evokes a systemic inflammatory response and increases the risk for cardiovascular disease [10–12]. Further, chronic infection and inflammation associated with periodontal disease are also thought to affect the pathogenesis of several types of cancer [21]. Moreover, the generation of carcinogens such as nitrosamines in periodontal disease is also suggested to increase the risk for cancer [10]. In recent years, several studies have reported that bacterial colonization in periodontitis may be associated with not just oral cancer, but also gastrointestinal tract cancers, such as in the colon or pancreas [38, 39]. However, the underlying mechanisms behind these associations are yet to be scientifically proven [40], warranting further studies in this regard.

This study could not confirm significant associations between the number of teeth and cancer-related mortality, which is an extremely controversial topic. Goto et al. surveyed the associations between number of teeth and cancer-related mortality, such as lung cancer, upper gastrointestinal cancer, and orodigestive cancer; significant associations were confirmed only between number of teeth and lung cancer [21]. Anzai et al. also reported similar results, confirming significant associations only between number of teeth and orodigestive cancer; the associations with other types of cancer (lung, stomach, pancreas, colon, and liver) were not significant [18]. The mechanism underlying the association between number of teeth and cancer-related mortality is unclear. However, as previously discussed, chronic infection, inflammation, generation of carcinogens, and bacterial colonization accompanying periodontitis have been advocated as risk factors for carcinogenesis. Considering the mechanisms reported in the previous study [18, 21], the oral health status, including the periodontal and oral hygiene status, should play a more significant role in cancer-related mortality than the number of teeth. Future studies should attempt to assess not just the number of teeth, but also the oral health status. Furthermore, it may also be necessary to survey site-specific cancer-related mortality.

Interestingly, having < 20 teeth was more strongly associated with all-cause mortality among the non-diabetics and non-smokers. In general, DM has various complications such as renal disease and macroangiopathy, which are known to increase mortality risk. The higher prevalence of other mortality risk factors in the diabetic participants may explain why the predictive power of tooth loss for mortality was diminished in these participants. On the contrary, the predictive power of tooth loss for mortality may be enhanced in the non-diabetics as they have a lower prevalence of other mortality risk factors than the diabetic participants. Similarly, in the non-smoking participants, the predictive power of tooth loss for mortality was enhanced due to the lower prevalence of other mortality risk factors in the non-smokers than in the smokers. As such, there is a possibility that if an individual is relatively healthy, such as a non-smoker or a non-diabetic, the number of teeth in the individual may have a significant impact on his/her survival. However, a subgroup analysis interpretation should be performed with caution [41, 42]. Occasionally, a low statistical power may be problematic due to the reduced number of participants in the subgroup analysis. Furthermore, there was a possibility of cognitive bias in this study; i.e., the number of non-diabetics may have been under-reported. Moreover, there may be unknown confounding factors, such as economic status. Further studies are required to confirm the effects of number of teeth on relatively healthy people.

Nevertheless, the present study had several limitations. First, we surveyed the number of teeth using a self-reported questionnaire; we did not confirm the number of teeth by clinical examination. Furthermore, we did not attempt to validate the correlation between the self-reported number of teeth and that determined by clinical examination. However, several reports have revealed that the number of teeth determined by self-reports and that determined by clinical examinations showed strong correlations [43, 44]. Thus, our methodology for confirming the number of teeth may not have influenced our results to a large extent. However, since a discrepancy between the self-reported number of teeth and that determined by clinical examination may exist, a validation for the correlation between the two counts should have been performed.

Second, we did not obtain detailed information on eating ability. A Japanese prospective cohort study surveyed the associations between oral health and cancer-, cardiovascular disease- and respiratory disease-related mortality [20]. That study showed significantly higher HRs for participants with ≤ 19 teeth and eating difficulty in comparison to those with ≥ 20 teeth. However, there were no significant HRs for participants with ≤ 19 teeth and those who could eat everything in comparison to those with ≥ 20 teeth. These results suggested that eating ability, rather than number of teeth, might affect respiratory disease- and cardiovascular disease-related mortality. These findings may elucidate our findings, which show a lack of significant association between < 20 teeth and cardiovascular disease-related mortality. Furthermore, another recent study suggested that the number of functional teeth was a stronger predictor for all-cause mortality than the total number of teeth among community-dwelling older adults [37]. We should have surveyed not just the number of teeth, but also obtained details on eating ability. Furthermore, we did not investigate oral hygiene and periodontal status either. As previously discussed, oral health status has a greater potential to affect mortality than the number of teeth, especially cancer-related mortality. A comprehensive survey on the oral cavity may be required in the future.

Third, the interpretation of results in cases wherein *p *value was close to 0.05. In our main findings regarding the association between the number of teeth and all-cause mortality, the CI of the HR for all-cause mortality was revealed to be very close to the null value (1.007), and the *p *value also approached non-significance. Therefore, the results must be interpreted with caution. *p *values should be considered as continuous variables rather than dichotomous ones (limited to only “significant” and “not significant”) for the purpose of demonstrating how often these observations would occur by chance. If *p* values are truly marginal, we need to take precautions to not overlook any clinically significant findings.

Fourth, was the methodology for surveying the participants’ educational status. The participants were categorized into three groups (low, middle, and high educational status) based on their age during their final educational qualification. Some participants might have failed their senior high school entrance examination or repeated some of their education in senior high school; therefore, a possibility of discrepancy in the participants’ true educational status cannot be denied. However, almost all the junior high school students in Japan subsequently go to high school, and most high school students graduate from high school at 18 years [24]. As such, the possibility of a discrepancy in the true educational status may not be problematic.

Fifth, the number of teeth in the participants may have changed over a period of time. There is a possibility that participants with < 20 teeth could have restored their teeth at any time during the follow-up period, changing their status from having < 20 teeth to ≥ 20 teeth through the use of prostheses, such as dental implants. There is also a possibility that participants with ≥ 20 teeth could have lost their teeth at any time during the follow-up period, changing their status from having ≥ 20 teeth to < 20 teeth. In this study, we only surveyed the number of teeth at baseline. Ideally, the change in the number of teeth should have been surveyed during the follow-up period as well.

Sixth, is selection bias. Our final participants may not be representative of the original target population, since 734 of 2942 participants were excluded due to incomplete data regarding their number of teeth. These excluded values may have affected the overall results.

Seventh, the participants’ smoking status was coded as binary variables; i.e., current smoker or non-smoker, which encompassed past smoking history. Several studies in the literature have indicated the importance of examining lifetime smoking history, rather than just examining the smoking history at a single point in time [45–48]. Smoking status should have been considered a continuous variable in the statistical analysis.

## Conclusions

Our study revealed that people with < 20 natural teeth have a significant risk of all-cause mortality, although the difference was borderline significant. However, the risk of cancer- and cardiovascular disease-related mortality did not reach statistical significance between the two groups. Our findings not only emphasize the importance of having ≥ 20 natural teeth for a healthy life expectancy, but also highlight the scope for further research in this field, with regards to data collection related to masticatory capacity, oral health status related to dental caries and periodontal disease, as well as a more detailed cause of mortality, especially cancer-related mortality.

## Data Availability

The raw data are confidential and cannot readily be shared. Researchers need to obtain permission from the Institutional Review Board and apply for access to the data from The Ethics Committee of Yamagata University.
